# Haematological Profile of Children With Sickle Cell Anaemia in Steady State

**DOI:** 10.7759/cureus.11011

**Published:** 2020-10-18

**Authors:** Rasaki Aliu, Jalo Iliya, Oladeji R Quadri, Olayinka R Ibrahim, Ezra Daniel

**Affiliations:** 1 Pediatrics, Gombe State University/Federal Teaching Hospital, Gombe, NGA; 2 Otorhinolaryngology, Gombe State University/Federal Teaching Hospital, Gombe, NGA; 3 Pediatrics, Federal Medical Center, Katsina, NGA; 4 Pediatrics, Federal Teaching Hospital, Gombe, NGA

**Keywords:** sickle cell children, haematological profile, iron markers, vascular occlusion

## Abstract

Introduction

Sickle cell anaemia (SCA) is an inherited, autosomal recessive condition that results from a mutation in the β-globin gene. Vascular occlusion is the underlying mechanism behind a myriad of complications encountered. This vascular occlusion is primarily caused by the increased tendency of red blood cells (RBC) to adhere to the vascular endothelium, and the activation of platelets and total leucocyte count (TLC), hence the need for a steady-state haematological profile in these patients.

Method

This was a cross-sectional study conducted over four months at a sickle cell clinic. Haemoglobin (Hb) concentration, haematocrit, platelet, TLC, mean corpuscular volume (MCV), mean corpuscular haemoglobin (MCH) and mean corpuscular haemoglobin concentration (MCHC) of the subjects were recorded and analysed.

Results

Ninety-nine subjects aged 1-18 years were recruited for the study. There were 53 (53.5%) males. Leucocytosis was seen in 80 (80.8%), anaemia in 99 (100%), and thrombocytosis in 30 (30.3%) patients. The mean Hb, TLC and platelets were 7.9 ± 1.3g/dl, 14.3 ± 4.5 x 10^3^/mm^3^ and 391.5 ± 182.6 x 10^3^/mm^3^ respectively. Mean MCV, MCH and MCHC were 81.3 ± 7.1 fl, 28.6 ± 2.9 pg and 35.2 ± 1.7 g/dl respectively. Children aged one to four years had the highest TLC (p=0.002) but the lowest mean Hb and platelet (p=0.094 and 0.06) respectively. The mean MCV, MCH and MCHC were lowest in children aged one to four years (p=0.047, 0.001 and 0.001).

Conclusion

Anaemia, leucocytosis and thrombocytosis are characteristics features of children with SCA, especially in male and younger subjects. Although Iron markers are generally normal in children with SCA, those under the age of five years tend to have lower values.

## Introduction

Globally, more than 300,000 babies with sickle cell disease are born each year [1]. More than two-thirds of these births occur in Africa, with Nigeria accounting for half (150,000) of the victims [1]. Although Nigeria as a country has the highest burden of sickle cell anaemia (SCA) in the world [1], most of the cases are reported in the northern region of the country, where this study was conducted, which has a prevalence rate of 23.7% [2].

SCA affects virtually every organ of the body [3]. While infection and vaso-occlusive and anaemic crises are common manifestations in children with SCA, vaso-occlusion has been reported as the most common manifestation and the most common cause of hospitalization, organ failure, and death in children with SCA [4]. This vaso-occlusion, which underlies many pathogenic mechanisms behind myriad complications in children with SCA, is primarily caused by adherence of red blood cells (RBC) to the vascular endothelium and thus anaemia, white blood cells (WBC) and platelet activation, leading to elevated values with potentials for developing complications [5]. Determining the steady-state values of these parameters and associated factors are therefore critical in detecting early deviations from the norm and prompt intervention. In this study, we describe the haematological indices of children with SCA in steady state. This study is the first of its kind to be conducted in North-East Nigeria, and we believe the findings from this study will add to the existing body of knowledge and form a reference template related to SCA for our environment and regional settings.

## Materials and methods

Study site

This was a cross-sectional descriptive study conducted at the sickle cell disease centre of the Federal Teaching Hospital, Gombe (FTHG), North-East Nigeria, and lasted four months from May to August 2018. The FTHG is a tertiary institution that has a sickle cell centre, which is one of the six sickle cell centres spread across the six geo-political zones in Nigeria.

Inclusion criteria

Children aged 1-18 years who were on follow-up at the sickle cell clinic in FTHG, in steady state (defined as the absence of fever and crisis in the previous four weeks or more in a child who was not on any medication other than routine folic acid and prophylactic antimalarial drug) were recruited after obtaining consent from their parents. Assent was also obtained from children aged seven years and above.

Exclusion criteria

Children who were on hydroxyurea were excluded as this would alter the steady-state haematological indices in SCA. Those who had received a blood transfusion within the previous month were also excluded.

Sampling technique

The researcher and the research assistant met with the staff of the sickle cell clinic a week before the commencement of the study and the information about the study was provided. Thereafter, the caregivers and the children were met weekly on their clinic days (Wednesday) early in the morning, and subjects were recruited consecutively after obtaining written informed consent, which included information about the study. Structured questionnaires were administered and 5 ml of venous blood samples were taken from the subjects for a complete blood count.

Ethical clearance

Ethical approval was obtained from the Research and Ethics committee (REC) of FTHG.

Data analysis

Data were processed and analysed using IBM SPSS Statistical software version 23.0 (IBM, Armonk, NY). The mean and standard deviations were computed for continuous variables such as haemoglobin (Hb), white blood cells (WBC) and platelet counts; the independent t-test was used to determine the level of statistically significant difference between two independent groups, and analysis of variance (ANOVA) was used for more than two groups. The categorical data such as sex, age groups, and social class were described using frequency and proportions while the Chi-square test was used to determine the level of the statistical differences. A p-value of <0.05 was considered statistically significant.

## Results

A total of 99 children with SCA in steady state were recruited for the study. The mean age of the males and females was 8.02 ± 5.11 and 10.35 ± 5.31 years respectively, and children aged five to nine years constituted the majority (28; 28.3%). There were 53 (53.5%) males and 46 (46.5%) females with a male-to-female ratio of 1.2:1. A higher proportion of the subjects (75; 75.8%) were from low socioeconomic classes. Table 1 shows the sociodemographic characteristics of the study subjects.

**Table 1:** Sociodemographic characteristics of children with sickle cell anaemia SES: socioeconomic status

Characteristic	Frequency	Percentage
Age (years)		
1-4	27	27.3
5-9	28	28.3
10-14	23	23.2
≥15	21	21.1
Gender		
Male	53	53.5
Female	46	46.5
SES		
Low	75	75.8
Middle	18	18.2
High	6	6.1

The mean TLC was higher in children under five years and male subjects, while their mean Hb and platelet were lower compared to other age groups and female subjects. All subjects (99; 100.0%) had anaemia. A total of 80 (80.8%) subjects had leucocytosis while none had leucopoenia. Thrombocytosis and thrombocytopenia were seen in 30 (30.3%) and seven (7.1%) subjects respectively (Table 2).

**Table 2:** Hb, TLC and platelet categorization in children with sickle cell anaemia* *[6] Hb: haemoglobin; TLC: total leucocyte count

Hb (n=99), n (%)	Total leucocyte count (n=99), n (%)			Platelet (n=99), n (%)		
	High	Normal	Low	High	Normal	Low
99 (100.0)	80 (80.8)	19 (19.2)	0 (0.0)	30 (30.3)	62 (62.6)	7 (7.1)

High TLC (leucocytosis) was more common (25; 25.3%) in subjects aged one to four years (under-five); the overall mean TLC was 14.3 ± 4.5 x 10^3^/mm^3^. The mean TLC was significantly higher in males (15.42 ± 5.11 x 10^3^/mm^3^) when compared to female subjects (12.98 ± 3.18 x 10^3^/mm^3^) (p=0.006). The mean TLC was higher in children under five years (17.03 ± 5.25 x 10^3^/mm^3^) compared to other age groups (p=0.002). The mean Hb was lower in males (7.63 ± 0.90 g/dL) compared to female subjects (p=0.038), and in subjects under five years (7.76 ± 1.17 g/dL) compared to other age groups (p=0.094). The mean corpuscular volume (MCV) was 81.26 ± 7.14 fl but was statistically significantly lower in under-five subjects (78.54 ± 5.92 fl) (p=0.047) compared to other age groups, and in male subjects (79.86 ± 6.98 fl) compared with females (82.88 ± 7.05 fl) (p=0.036). The mean corpuscular haemoglobin (MCH) was 28.63 ± 2.90 pg. The under-five subjects had a statistically significant lower value (26.79 ± 2.53 pg) compared to other age groups (p=<0.001). The male participants had significantly lower MCH (27.96 ± 3.09 pg) compared with the females (p=0.012). The mean corpuscular haemoglobin concentration (MCHC) was 35.18 ± 1.68 g/dL and was significantly lower in under-five children (34.04±1.59 g/dL) compared with other age groups (p<0.001). The mean MCHC was lower in male subjects (34.91 ± 1.81 g/dL), but the difference was not significant (p=0.088) (Tables 3, 4).

Thrombocytosis and thrombocytopenia were seen in 30 (30.3%), and seven (7.1%) subjects respectively. The mean platelet count was 391.5 ± 182.6 x 10^3^/mm^3^. The mean platelet count was lowest in under-five children, who had a mean value of 350.48 ± 134.73 x 10^3^/mm^3^, when compared with other age groups (p=0.060). The platelet was higher in male subjects, who had a mean platelet of 400.67 ± 200.23 x 10^3^/mm^3^ (Tables 3, 4).

**Table 3:** Haematological profile of children with sickle cell anaemia based on age group Hb: haemoglobin; MCV: mean corpuscular volume; MCH: mean corpuscular haemoglobin; MCHC: mean corpuscular haemoglobin concentration; TLC: total leucocyte count

Variables	1-4 years	5-9 years	10-14 years	15-18 years	P-value	Overall
Hb (g/dl)	7.66 ± 1.08	7.76 ± 1.19	7.72 ± 1.11	8.50 ± 1.64	0.094	7.89 ± 1.27
HCT (%)	21.56 ± 2.99	22.89 ± 3.28	21.80 ± 2.90	23.52 ± 4.75	0.189	22.41 ± 3.52
MCV (fl)	78.57 ± 5.92	80.92 ± 8.22	82.17 ± 6.90	84.20 ± 6.37	0.047	81.26 ± 7.14
MCH (pg)	26.80 ± 2.53	28.44 ± 2.82	29.38 ± 2.26	30.44 ± 2.81	0.001	28.63 ± 2.90
MCHC (g/dl)	34.04 ± 1.59	35.18 ± 1.87	35.79 ± 1.24	35.98 ± 1.10	0.001	35.18 ± 1.68
TLC (10^3^/mm^3^)	17.03 ± 5.26	13.77 ± 4.26	12.74 ± 3.55	13.16 ± 3.04	0.002	14.29 ± 4.47
Platelet (10^3^/mm^3^)	350.5 ± 134.7	457.3 ± 240.3	371.6 ± 156.8	378.1 ± 160.8	0.060	391.5 ± 182.6

**Table 4:** Haematological profile of children with sickle cell anaemia based on gender Hb: haemoglobin; MCV: mean corpuscular volume; MCH: mean corpuscular haemoglobin; MCHC: mean corpuscular haemoglobin concentration; TLC: total leucocyte count

Investigation	Males	Females	P-value
Hb (g/dl)	7.63 ± 0.90	8.16 ± 1.56	0.027
HCT (%)	21.86 ± 2.53	23.03 ± 4.32	0.100
MCV (fl)	79.86 ± 6.98	82.88 ± 7.05	0.036
MCH (pg)	27.96 ± 3.09	29.42 ± 2.48	0.012
MCHC (g/dl)	34.91 ± 1.81	35.49 ± 1.47	0.088
TLC (10^3^/mm^3^)	15.43 ± 5.11	12.98 ± 3.18	0.006
Platelet (10^3^/mm^3^)	400.67 ± 200.23	380.848 ± 150.99	0.593

## Discussion

In this study, leucocytosis (elevated TLC) was a characteristic finding in children with SCA; it was seen in more than two-thirds of the subjects. This finding is similar to the reports of other studies [7,8]. The leucocytosis in SCA at steady state has been hypothesised to be a reflection of the constant inapparent inflammation with consequent cytokine release ultimately leading to increased leucocyte production in the bone marrow [9]. In this study, the mean leucocytosis was significantly higher in subjects who were less than five years of age compared to older children. Similar findings have been documented by West et al. [10]. The higher mean leucocytosis in under-five children with SCA may be due to an increased incidence of covert and overt infections that occur in younger children [11]. This may also explain the increased mortality in young children with SCA as leucocytosis has been shown to be a significant risk factor for early death in SCA [12].

In this study, the mean leucocytosis was significantly higher in male subjects compared to females. This is similar to findings by Abubakar et al. and Iheanacho et al. who have also reported higher TLC values in male subjects with SCA [7,8]. Elevated platelet count (thrombocytosis) was seen in one-third of the children with SCA. This is consistent with findings relating to thrombocytosis that have been reported by other similar studies [7,8,13]. Thrombocytosis in SCA has been variably attributed to anaemia-induced increased erythropoietin secretion that has similar homology to thrombopoietin with consequent thrombopoiesis and decreased pooling of platelets in splenic circulation due to functional and/or structural asplenia characteristic of SCA [14,15]. The mean platelet was lowest in under-five subjects in the current study. This might be due to relatively intact splenic function and structure in the younger age group, causing platelets to be largely pooled in the spleen, unlike older children whose spleen have undergone recurrent infarction that characterises SCA. However, this finding contrasts with Iheanacho’s report, who adduced the observed decreased platelet with age to reduced thrombopoietin in adults [8]. However, this may not be the case as platelet count in SCA is dependent on more than one factor [14,15]. This will probably explain the significantly higher platelet count in male subjects in the current study. The males were albeit younger and thus lower platelet count was expected; their higher degree of anaemia might have caused the elevated platelet [15]. Thus, in children with SCA, the lower platelet count is dependent on younger age, a higher degree of anaemia, among other factors.

All subjects (100%) had low Hb in this study. Other studies have shown that low Hb in children with SCA is caused by reduced RBC life span (one-seventh of normal), low erythropoietin response and nutritional deficiency [10,16,17]. These would explain the low Hb in all the subjects as more than two-thirds were from low socioeconomic class. This finding is similar to other reports [7,8]. The mean Hb was lowest in under-five subjects and younger male subjects in this study. The low Hb in younger subjects is similar to the reports by Iheanacho et al. [8], while the low Hb in males is similar to the report by Abubakar et al. [7]. Iheanacho et al. [8] and Rao et al. [18] have reported higher Hb in males, although they were not statistically significant. The younger age of male subjects and the predominantly low socioeconomic status of under-five subjects in the current study might explain the observed low mean Hb [17,19]. The mean MCV, MCH and MCHC, which are the markers of iron status, were all within normal ranges [20]. This finding is similar to other reports [7,21,22]. The normal iron markers in children with SCA may be attributed to increased red cell turnover and repeated blood transfusion. The values in under-five and male subjects were lowest in this study, probably due to age variability factor and low socioeconomic status [17,19].

## Conclusions

Leucocytosis, thrombocytosis, low Hb and normal iron markers (MCV, MCH, MCHC) are the characteristic features in children with SCA, and these features are predominantly found among children under five years of age compared to other age groups. Close monitoring of under-five children with SCA is thus highly recommended.
